# The antioxidant compound tert-butylhydroquinone activates Akt in myocardium, suppresses apoptosis and ameliorates pressure overload-induced cardiac dysfunction

**DOI:** 10.1038/srep13005

**Published:** 2015-08-11

**Authors:** Yongtao Zhang, Fang Fang Liu, Xiaolei Bi, Shuangxi Wang, Xiao Wu, Fan Jiang

**Affiliations:** 1 Key Laboratory of Cardiovascular Remodeling and Function Research, Qilu Hospital; 2 Department of Pathophysiology, School of Medicine, Shandong University, Jinan, Shandong Province, China

## Abstract

Tert-butylhydroquinone (TBHQ) is an antioxidant compound which shows multiple cytoprotective actions. We evaluated the effects of TBHQ on pathological cardiac remodeling and dysfunction induced by chronic overload. Pressure overload was created by transverse aortic constriction (TAC) in male C57BL/6 mice. TBHQ was incorporated in the diet and administered for 4 weeks. TBHQ treatment prevented left ventricular dilatation and cardiac dysfunction induced by TAC, and decreased the prevalence of myocardial apoptosis. The beneficial effects of TBHQ were associated with an increase in Akt activation, but not related to activations of Nrf2 or AMP-activated protein kinase. TBHQ-induced Akt activation was accompanied by increased phosphorylation of Bad, glycogen synthase kinase-3β (GSK-3β) and mammalian target of rapamycin (mTOR). Mechanistically, we showed that in cultured H9c2 cells and primary cardiac myocytes, TBHQ stimulated Akt phosphorylation and suppressed oxidant-induced apoptosis; this effect was abolished by wortmannin or an Akt inhibitor. Blockade of the Akt pathway *in vivo* accelerated cardiac dysfunction, and abrogated the protective effects of TBHQ. TBHQ also reduced the reactive aldehyde production and protein carbonylation in stressed myocardium. We suggest that TBHQ treatment may represent a novel strategy for timely activation of the cytoprotective Akt pathway in stressed myocardium.

Heart failure is a debilitating disease associated with high morbidity and mortality, and with increased healthcare costs^1^. Under stress conditions such chronic overload or myocardial infarction, cardiac myocytes initiate a hypertrophic response, which is thought to be an adaptive reaction and have compensatory effects on cardiac pumping functions; however, the presence of persistent stress stimuli will eventually result in myocardial decompensation, which is associated with ventricular dilatation, myocardial fibrosis, oxidative stress, contractile dysfunction and finally heart failure^2^,^3^,^4^. Mechanisms underlying the transition from adaptive hypertrophy to heart failure are poorly understood. Several lines of evidence have suggested that loss of cardiac myocytes in the form of apoptosis and/or necrosis, abnormalities in intracellular calcium homeostasis, and uncontrolled fibrosis in the myocardium may all have essential roles in this pathological process, and these may represent potential therapeutic targets to prevent the development heart failure^2^,^3^,^5^. Despite the intense research in this area, currently we still lack an effective pharmacological approach that can block the progression of myocyte decompensation and heart failure^1^,^5^,^6^.

Tert-butylhydroquinone (TBHQ) is a phenolic chain-breaking antioxidant used to prevent lipid peroxidation^7^. Several *in vivo* studies showed that TBHQ treatment elicited significant cytoprotective actions in different organs under pathological conditions. For example, systemic or local intra-cerebroventricular treatment with TBHQ in an ischemic stroke model in rats significantly reduced the infarct size and neurological deficits^8^. Similarly, there was evidence showing that administration of TBHQ in rats suppressed renal damage and oxidative stress after ischemia and reperfusion injury^9^. In mice with type 1 diabetes, chronic treatment with TBHQ significantly reduced the degree of glomerular fibrosis and ameliorated proteinuria^10^. TBHQ also showed prominent neuro-protective actions in both of experimental traumatic brain injury and Alzheimer’s disease models^11^,^12^. Recently, Lin *et al.* reported echocardiographic data suggesting that TBHQ might also have beneficial effects in the heart under chronic pressure overload condition^13^. Nonetheless, the pharmacological effects of TBHQ on the histopathological and molecular changes in stress-induced cardiac remodeling remain unknown. Moreover, the mechanisms of the potential cardiac protective action of TBHQ are still poorly understood.

TBHQ is an activator of nuclear factor erythroid 2-related factor 2 (Nrf2), a redox-sensitive transcription factor with multiple cytoprotective functions, including in cardiomyocytes^14^,^15^,^16^. On the other hand, numerous studies have shown that activation of the Akt pathway have crucial protective effects against the development of myocardial dysfunction and failure induced by chronic overload^17^,^18^,^19^,^20^,^21^,^22^,^23^,^24^. Interestingly, there is evidence indicating that TBHQ may stimulate the activation of the Akt pathway^25^, whereas it is unknown whether TBHQ has a similar effect in cardiomyocytes. In this report, we presented evidence showing that TBHQ inhibited myocyte apoptosis and prevented the development of ventricular dilatation and dysfunction induced by chronic overload. Our data suggest that acute activation of Akt, but not the Nrf2 pathway, may have a pivotal role in mediating the anti-apoptotic and cardiac protective actions of TBHQ in stressed hearts.

## Results

### TBHQ prevented TAC-induced ventricular dilatation and dysfunction

**Figure 1:**
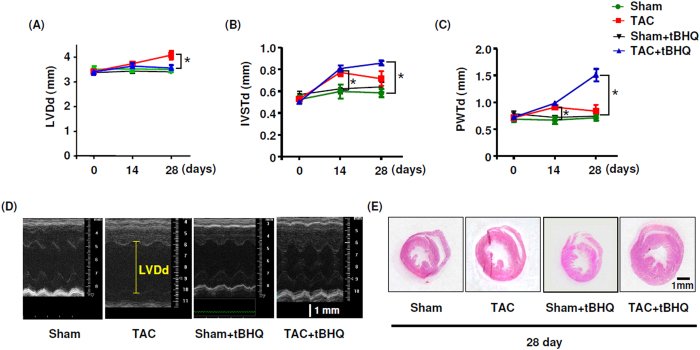
Effects of TBHQ treatment on TAC-induced cardiac remodeling. (**A**–**C**) Echocardiography data showing changes in left ventricle end-diastolic diameter (LVDd), inter-ventricular septum end-diastolic thickness (IVSTd) and posterior wall end-diastolic thickness (PWTd) in sham, TAC, sham + TBHQ and TAC + TBHQ groups. (**D**) Original M-mode echocardiography images of heats from different groups. (**E**) Horizontal short axis plane sections of heart showing gross changes in the ventricular dimension and wall thickness in different groups. Data are mean ± SEM. *P < 0.05, one-way ANOVA, *n* = 5 (Sham), 6 (Sham + TBHQ), 12 (TAC) and 12 (TAC + TBHQ).

**Figure 2:**
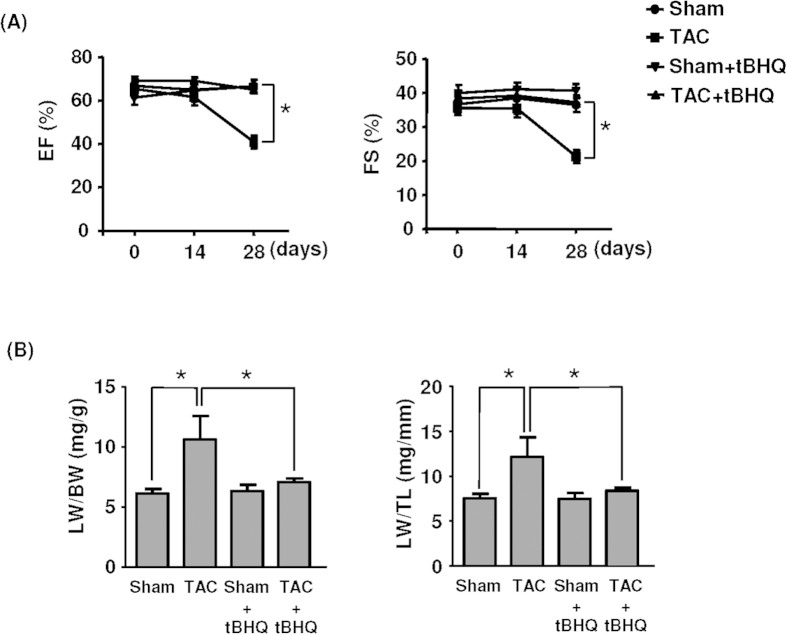
Effects of TAC and TAC + TBHQ on (**A**) ejection fraction (EF) and fractional shortening (FS) and (**B**) lung weight (LW) to body weight (BW) ratio and LW to tibia length (TL) ratio. Data in (**B**) were obtained at 4 weeks after TAC. *P < 0.05, one-way ANOVA, *n* = 5 (Sham), 6 (Sham + TBHQ), 12 (TAC) and 12 (TAC + TBHQ).

Chronic treatment with TBHQ did not show any toxic effects as evidenced by the stable body weight or the general parameters for liver and kidney functions (Table 1). Neither TAC nor TBHQ had significant effects on food and water consumptions. We characterized the time course of pressure overload-induced cardiac remodeling under the present experimental settings; we found that at 2 weeks, there was a significant LV wall thickening, but no LV dilatation (Fig. 1A–C). At 4 weeks, however, there was a notable LV dilatation, indicating a status of myocardial decompensation. We observed that TBHQ treatment prevented the occurrence of LV dilatation induced by TAC (Fig. 1A–E). Consistent with the above findings, we demonstrated that the ejection fraction (EF) and FS were not different between sham and TAC groups at 2 weeks, whereas EF and FS were significantly decreased in TAC animals at 4 weeks (Fig. 2A). TBHQ treatment restored the EF and FS values in TAC animals (Fig. 2A). Moreover, we found that the weight of lungs was significantly elevated in TAC animals at 4 weeks, indicating a sign of congestive heart failure. This change was similarly prevented by TBHQ treatment (Fig. 2B). We also measured the hepatic content of hemoglobin (passive liver congestion) as an additional sign of congestive heart failure. Using Drabkin’s reagent (Sigma), we showed that the hepatic hemoglobin content was significantly elevated in the TAC group as compared to sham (36.2 ± 2.9 versus 69.4 ± 10.0 μg/mg tissue, *P* < 0.05), and this change was attenuated by TBHQ (46.6 ± 6.2 μg/mg, *P* < 0.05 versus TAC, one-way ANOVA, *n* = 5). In sham animals, TBHQ treatment alone did not produce significant changes in the geometry of LV or the myocardial contractility (Figs 1 and 2). These data are in agreement with those reported by others^13^.

### Effects of TBHQ on myocardial hypertrophy and fibrosis

**Figure 3:**
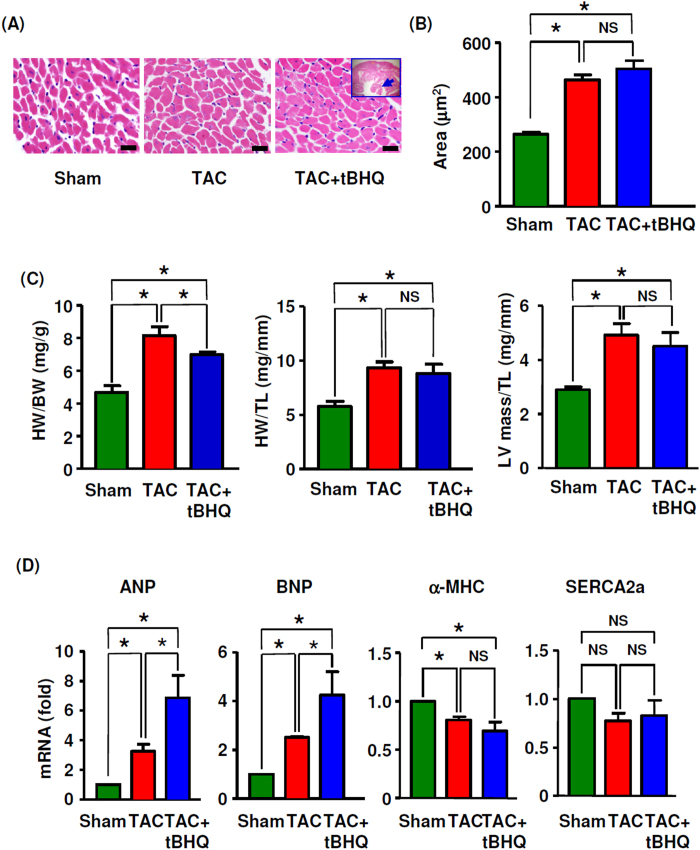
Effects of TBHQ on TAC-induced myocyte hypertrophy. (**A**) Hematoxylin and eosin stained sections of left ventricular myocardium (bar = 20 μm). All sections were obtained at the same short axis plane as indicated by the insert; the arrow indicated the papillary muscle. (**B**) Mean cross sectional areas of muscle fibers (*n* = 5 animals). (**C**) Changes in heart weight (HW) to body weight (BW) ratio, HW to tibia length (TL) ratio and echo-derived LV mass to TL ratio, *n* = 5 (Sham), 12 (TAC) and 12 (TAC + TBHQ). (**D**) Real-time PCR results showing the expression levels of atrial natriuretic peptide (ANP), B-type natriuretic peptide (BNP), α-myosin heavy chain (MHC) and sarcoplasmic reticulum Ca^2+^ ATPase-2a (SERCA2a) in the heart (*n* = 6 each). *P < 0.05, one-way ANOVA. NS, no significance.

**Figure 4:**
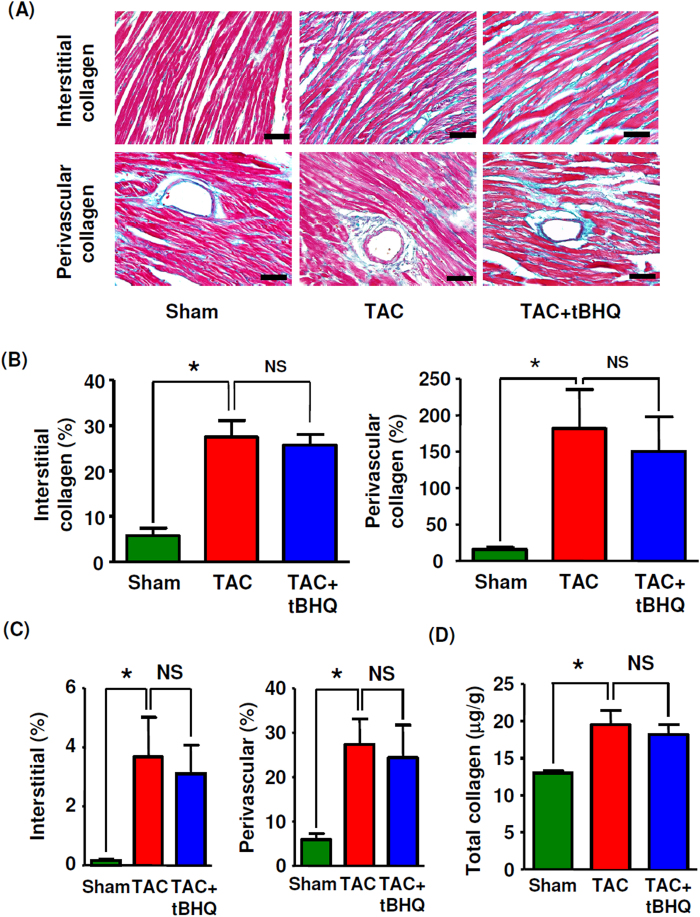
Effects of TBHQ on TAC-induced cardiac fibrosis. (**A**) Masson’s trichrome staining showing interstitial and perivascular deposition of collagen fibers (blue color) (400 × images, bar = 50 μm). (**B**) Quantitative data of Masson’s trichrome staining for collagen deposition in LV (expressed as % area of the whole section for interstitial or as % area of the vessel lumen for perivascular) (*n* = 5–8 animals). (**C**) Quantitative data of picrosirius red staining for collagen deposition in LV (*n* = 5–8 animals). (**D**) LV collagen contents measured by hydroxyproline assay (*n* = 6 animals). *P < 0.05, one-way ANOVA. NS, no significance.

Next we examined overload-induced pathological changes in the myocardium and the effects of TBHQ at 4 weeks. Morphometric analysis revealed that TAC resulted in typical myocyte hypertrophy. However, treatment with TBHQ showed no significant effect on TAC-induced myocyte hypertrophy (Fig. 3A,B). Consistently, the echocardiography data also showed that TBHQ treatment maintained a hypertrophic phenotype of the ventricular wall throughout the period of TAC (Fig. 1B,C). The heart to body weight ratio, the heart weight to tibia length ratio and the echo-derived LV mass to tibia length ratio significantly increased in the TAC group at 4 weeks. TBHQ treatment had no significant effects on the heart weight to tibia length ratio or the echo-derived LV mass to tibia length ratio, while it partially reduced the heart to body weight ratio (Fig. 3C). We proposed that the observed decrease in the heart to body weight ratio could be caused by the moderate increase in body weight in TBHQ treated animals (see Table 1). TAC significantly upregulated the expression levels of atrial natriuretic peptide and B-type natriuretic peptide, markers of pathological hypertrophy; however, treatment with TBHQ further increased the natriuretic peptide levels (Fig. 3D). Moreover, TBHQ had no significant effects on the expression levels of α-myosin heavy chain or sarcoplasmic reticulum Ca^2+^ ATPase-2a (Fig. 3D). We also assessed changes in myocardial fibrosis using Masson’s trichrome staining, picrosirius red staining and hydroxyproline assay. We found that TBHQ had no significant effects on the severity of TAC-induced fibrosis (Fig. 4A–D and Supplemental Figure S1). Collectively, the effects of TBHQ on hypertrophy or fibrosis cannot explain its beneficial effects on TAC-induced cardiac dysfunction.

### TBHQ did not activate Nrf2 or AMPK in the myocardium

**Figure 5:**
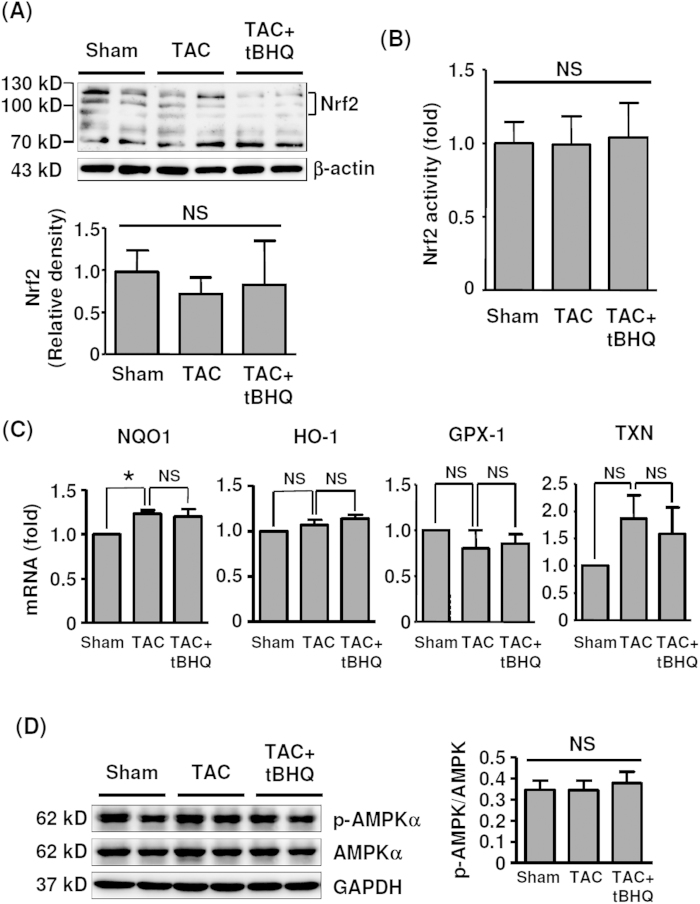
Effects of TAC and TBHQ on Nrf2 and AMPK pathways in the heart. (**A**) Western blot and quantitative densitometry data showing the protein levels of Nrf2 in the LV myocardium (*n* = 6). (**B**) Nrf2 DNA binding activity measured in the nuclear protein extracts of myocardium (*n* = 6). (**C**) PCR results showing expression levels of NQO1, HO-1, glutathione peroxidase-1 (GPX-1) and thioredoxin (TXN) (*n* = 6). (**D**) Western blot and quantitative densitometry data showing the levels of phospho- and total AMPKα (*n* = 6). *P < 0.05, one-way ANOVA. NS, no significance; GAPDH, glyceraldehyde-3-phosphate dehydrogenase.

We measured the endogenous levels of Nrf2 in LV homogenates at 4 weeks. As reported by other laboratories^26^, we observed that the Nrf2 antibody detected multiple bands (Fig. 5A). It has been determined that endogenous Nrf2 runs as 95–110 kDa bands in SDS-PAGE, rather than the predicted ~65 kDa band^26^. Surprisingly, under current experimental setting, TBHQ failed to stimulate Nrf2 accumulation in the myocardium (Fig. 5A). To confirm this finding, we directly measured the Nrf2 DNA binding activity of the nuclear protein extracts of LV, using a TransAM kit from Active Motif (Carlsbad, CA, USA). We showed that there were no significant changes in Nrf2 activity in different groups (Fig. 5B). In addition, we measured expression levels of the Nrf2 target genes NAD(P)H:quinone oxidoreductase 1 (NQO1), heme oxygenase-1 (HO-1), glutathione peroxidase-1 and thioredoxin; we found that TBHQ did not significantly change the mRNA levels of these genes, although TAC *per se* increased the NQO1 expression (Fig. 5C). To exclude the possibility that the lack of Nrf2 response to TBHQ in the myocardium was an artifact, we examined Nrf2 target genes in the liver and kidney. We showed that in these organs, the expressions of NQO1 and HO-1 were all significantly elevated in TBHQ-treated animals (Supplemental Figure S2). Also, we demonstrated that the phosphorylation level of AMPKα was not affected by TBHQ (Fig. 5D). Moreover, TBHQ exhibited no significant effects on expression levels of the ER stress markers CHOP and GRP78 (data not shown).

### TBHQ inhibited myocardium apoptosis via increased Akt phosphorylation

**Figure 6:**
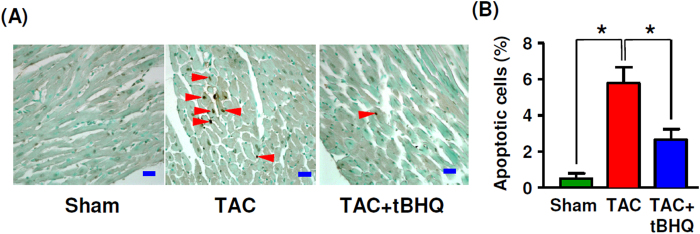
Effects of TAC without and with TBHQ treatment on myocyte apoptosis. (**A**) Images of TUNEL staining showing apoptotic cells (arrowheads) in LV (bar = 20 μm). (**B**) Count of apoptotic cells (expressed as % of the total nuclei counted) in different groups. *P < 0.05, one-way ANOVA (*n* = 5 animals).

**Figure 7:**
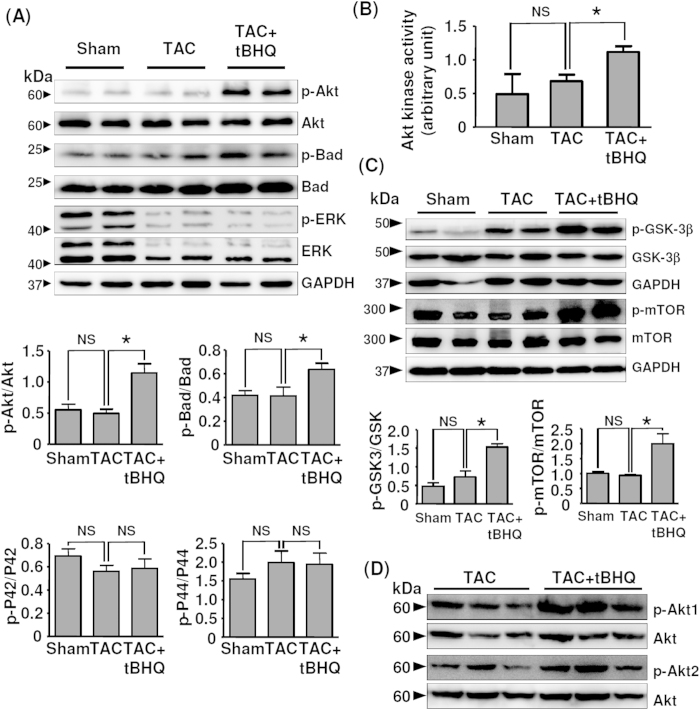
TBHQ activated the Akt pathway in the heart *in vivo*. (**A**) Western blot and quantitative densitometry data showing the levels of phosphorylated and total Akt, Bad and ERK1/2 in LV (*n* = 6). (**B**) Akt kinase activity measured in the myocardium (*n* = 6). (**C**) Western blots showing the effect of TBHQ on phosphorylation of glycogen synthase kinase-3β (GSK-3β) and mammalian target of rapamycin (mTOR). (**D**) Western blots showing the effects of TBHQ on phosphorylation of Akt1 and Akt2 in the myocardium. *P < 0.05, one-way ANOVA.

TUNEL assays in LV sections at 4 weeks demonstrated that TAC induced ~ 10-fold increase in myocyte apoptosis as compared to sham animals. TBHQ treatment significantly suppressed TAC-induced apoptosis by about 60% (Fig. 6A,B). There is evidence suggesting that TBHQ may affect the activation of Akt in cancer cell lines^25^. Therefore we examined whether TBHQ modulated Akt phosphorylation in the myocardium. As shown in Fig. 7A, we found that in TBHQ-treated hearts, there was a significant increase in the phosphorylation level of Akt as compared to sham and TAC animals. In accordance with the enhanced Akt phosphorylation, we observed that the phosphorylation level of Bad, an anti-apoptotic protein and established Akt substrate, was also increased in TBHQ-treated hearts. In contrast, phosphorylation of ERK1/2 was not affected by TBHQ, albeit the total amount of ERK1/2 appeared to be downregulated in TAC-treated hearts. To further confirm the activation of the Akt pathway by TBHQ, we directly measured the Akt kinase activity using an assay kit from Cell Signaling Technology. As shown in Fig. 7B, Akt activity was significantly increased in TBHQ-treated hearts. Moreover, we showed that the phosphorylation level of the endogenous Akt substrates glycogen synthase kinase-3β and mammalian target of rapamycin (mTOR) were also increased by TBHQ (Fig. 7C). To clarify whether the effect of TBHQ on Akt activation was isoform specific, we measured the phosphorylation levels of Akt1 and Akt2, and found that phosphorylation of both of Akt1 and Akt2 was increased in TBHQ-treated hearts (Fig. 7D).

**Figure 8:**
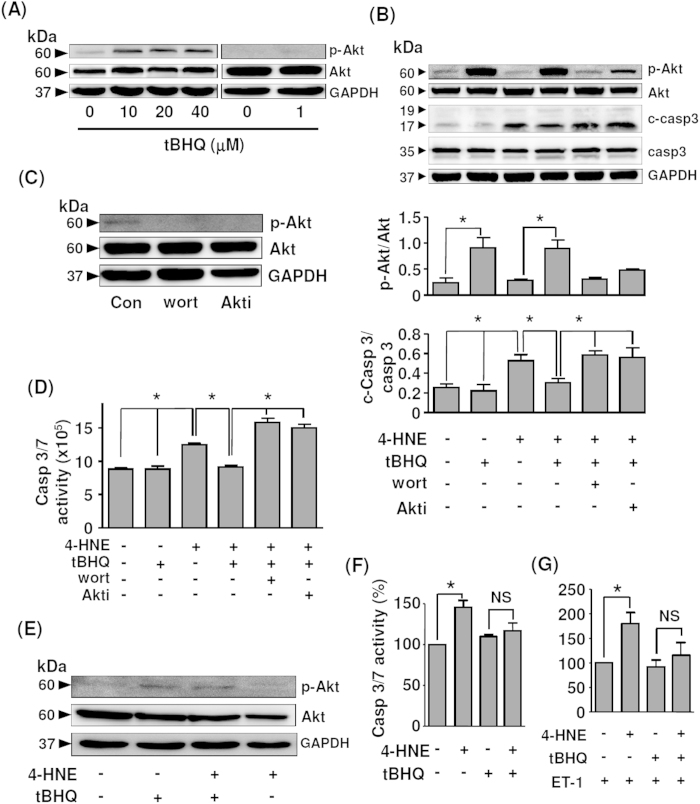
Effects of TBHQ on Akt activation and cell apoptosis *in vitro*. (**A**) Effects of TBHQ treatment at different concentrations for 24 hr on Akt phosphorylation in cultured H9c2 cells. (**B**) Effects of TBHQ (20 μM), wortmannin (10 nM) and Akt Inhibitor (Akti) (10 μM) pretreatments on Akt phosphorylation and 4-HNE (20 μM for 24 hr)-induced caspase 3 cleavage in H9c2 cells. c-Casp3, cleaved form of caspase 3. (**C**) Effects of wortmannin and Akt Inhibitor on the basal level of Akt phosphorylation. (**D**) Effects of TBHQ, wortmannin and Akti on 4-HNE-induced caspase 3/7 activation in H9c2 cells (expressed as arbitrary luminescence intensity). (**E**) Western blot showing the effects of TBHQ (20 μM for 12 hr) on Akt phosphorylation in the absence or presence of 4-HNE (20 μM) in rat primary cardiac myocytes. (**F**) Effects of TBHQ on 4-HNE-induced apoptosis in rat primary myocytes as measured by caspase 3/7 activity. (**G**) Effects of TBHQ on 4-HNE-induced apoptosis in rat primary myocytes in the presence of endothelin-1 (ET-1, 100 nM). *P < 0.05, one-way ANOVA (*n* = 3–4 independent experiments).

To clarify whether TBHQ could inhibit myocyte apoptosis via Akt activation, we treated H9c2 cells with TBHQ at different concentrations. We confirmed that TBHQ at concentrations up to 50 μM did not show cytotoxic effects in H9c2 cells. As shown in Fig. 8A and Supplemental Figure S3, TBHQ from 10 to 40 μM increased Akt phosphorylation, while TBHQ at 1 μM showed no effect. In the following experiments, we treated the cells with TBHQ at 20 μM as used by others^8^,^27^,^28^. 4-HNE is a reactive aldehyde produced during membrane lipid peroxidation with known apoptosis-inducing effects^29^,^30^. In our preliminary experiments, we found that 4-HNE produced more stable apoptotic responses in H9c2 than H_2_O_2_. Here we demonstrated that TBHQ suppressed 4-HNE-induced caspase 3 cleavage, which was accompanied by concomitant augmentation in Akt phosphorylation (Fig. 8B). We further demonstrated that the anti-apoptotic effect of TBHQ was abolished in the presence of wortmannin or Akti (Fig. 8B). Wortmannin and Akti also reduced the basal level of Akt phosphorylation (Fig. 8C). To confirm the results of caspase 3 western blotting, we directly measured the activity of caspase 3/7 and revealed that TBHQ treatment suppressed 4-HNE-elicited caspase 3/7 activation, while this effect of TBHQ was reversed by wortmannin or Akti (Fig. 8D). In addition, we performed TUNEL staining in cultured H9c2 cells and demonstrated similar anti-apoptotic actions of TBHQ (Supplemental Figure S4). As the H9c2 cell line might not precisely reflect the biology of mature myocytes, we further tested the effects of TBHQ in neonatal rat primary cardiac myocytes. We showed that TBHQ treatment increased Akt phosphorylation in the absence or presence of 4-HNE (Fig. 8E). Similar to H9c2 cells, TBHQ also suppressed 4-HNE-induced apoptosis in primary myocytes (Fig. 8F). Moreover, we confirmed that the anti-apoptotic effects of TBHQ in primary myocytes were preserved in the presence of the pro-hypertrophic agonist endothelin-1 (Fig. 8G).

To further confirm the role of the Akt pathway in mediating TBHQ effects *in vivo*, we co-treated the animals with LY294002 (3 mg/kg per day, i.p.) as described in previous studies^31^,^32^. We found that blockade of the Akt pathway changed the time course of cardiac dysfunction. Significant decreases in LV contractile functions and increase in the lung weight were detected at 2 weeks in LY294002-treated animals (Supplemental Figure S5). LY294002 also partially inhibited the development of hypertrophy. Importantly, in the presence of LY294002 co-treatment, TBHQ showed no significant beneficial effects on TAC-induced cardiac dysfunctions (Supplemental Figure S5). We also assessed the impacts of LY294002 on the apoptotic processes. We found that at 2 weeks, the endogenous Akt phosphorylation level was enhanced in TAC animals (in contrast to that at 4 weeks), likely representing an acute compensatory mechanism (Supplemental Figure S6). TBHQ did not further increase this response significantly. Moreover, we found that the level of apoptosis was not significantly different between sham and TAC at 2 weeks, which was in agreement with the functional data shown in Fig. 2A. However, we found that concomitant LY294002 treatment abrogated TAC-induced Akt activation at 2 weeks, and significantly increased the level of apoptosis (Supplemental Figure S6). Moreover, we showed that TBHQ had no significant effects on either Akt phosphorylation or apoptosis in the presence of LY294002 (Supplemental Figure S6). In addition, we also tested whether mTOR was involved in TBHQ-induced cardiac protection, as Akt activation by TBHQ was accompanied by increased mTOR phosphorylation. We co-treated animals with the mTOR inhibitor rapamycin (2 mg/kg per day, i.p.) as described previously^33^,^34^; we found that like LY294002, rapamycin also partially inhibited the development of hypertrophy at 2 weeks (IVSTd and PWTd being 0.79 ± 0.04 and 0.72 ± 0.09 in rapamycin-treated versus 1.1 ± 0.05 and 1.0 ± 0.04 in TAC animals respectively, all *P* < 0.05, n = 6). Consistent with previous reports^33^,^35^,^36^, we found that at 4 weeks, rapamycin co-treatment improved the FS and EF values in TAC animals (20.5 ± 1.3 and 36.8 ± 2.3 in TAC; 30.6 ± 1.7 and 50.4 ± 4.1 in TAC + rapamycin (all *P* < 0.05 versus TAC). However, we found that TBHQ still showed further beneficial effects on LV functions in the presence of rapamycin (38.7 ± 2.6 and 68.2 ± 3.8 in TAC + rapamycin + TBHQ animals (all *P* < 0.05 versus TAC + rapamycin).

### TBHQ reduced aldehyde production and protein carbonylation in myocardium

To clarify whether TBHQ exerted any antioxidant effects in the cardiac tissue, we performed immunohistochemistry for 4-HNE in samples of 4 weeks. Supplemental Figure S7A showed that 4-HNE immunoreactivity displayed a patchy distribution pattern. TAC significantly increased 4-HNE production in the myocardium, which was reduced by TBHQ treatment. Using SDS-PAGE and western blotting, we demonstrated that the abundance of carbonylated proteins in the myocardium was significantly raised by TAC, and TBHQ treatment suppressed TAC-induced accumulation of protein carbonyls (Figure S7B & C).

## Discussion

The major finding in this study was that TBHQ prevented the development of LV dilatation and systolic dysfunction induced by chronic pressure overload. These effects are consistent with the observations reported recently by Lin *et al.*^13^. However, we found that TBHQ had no significant effects on the process of TAC-induced myocyte hypertrophy or fibrosis, suggesting that the cardiac protective actions produced by TBHQ involved other mechanisms. Accumulating evidence has suggested that myocyte loss as a consequence of cell apoptosis and/or necrosis may have a pivotal role in the process of transition from myocardial hypertrophy to functional decompensation and failure^2^,^3^,^5^,^37^. In line with this notion, our results revealed that TBHQ treatment significantly reduced the prevalence of myocyte apoptosis in TAC-treated hearts. Taken these together, our data suggest that TBHQ treatment decelerates transition from compensated cardiac hypertrophy to heart failure, particularly through inhibition of myocyte apoptosis.

In response to cellular stress, activated Nrf2 mediates expression of an array of cytoprotective enzymes including NQO1, glutathione peroxidase, HO-1, and superoxide dismutase^15^. Although TBHQ is a well documented Nrf2 activator, we observed that the cardiac protective actions of TBHQ were not associated with changes in either Nrf2 protein accumulation or Nrf2 activation. Consistently, TBHQ did not significantly change the expressions of various Nrf2 target genes. This finding is in contrast to those obtained in the central nervous system and kidney, which indicated a critical role of Nrf2 activation in TBHQ-induced cytoprotection^8^,^10^. It is not clear why TBHQ was ineffective in the heart to activate the Nrf2 pathway, although we clearly showed that the current treatment regime readily stimulated NQO1 and HO-1 expressions in both of liver and kidney. In addition, there is evidence showing that TBHQ may activate the AMPK pathway^38^, which also has important protective functions in the heart^39^. However, our data suggest that the observed cardiac protective actions of TBHQ are unlikely to be related to activation of the AMPK pathway. Moreover, TBHQ did not have an impact on the ER stress response in stressed cardiac myocytes (data not shown).

Some pro-survival signaling pathways, such as ERK1/2, glycogen synthase kinase-3β and Akt, are downregulated during the progression of myocardial decompensation, raising the possibility that these pathways may be functional targets in the prevention of myocyte apoptosis under stress conditions^40^. In the present study, we also observed that the total ERK1/2 level was reduced in TAC-treated hearts. However, TBHQ showed no effects on ERK1/2 expression or phosphorylation. In stead, we demonstrated that there were significant increases in Akt phosphorylation and activity after TBHQ treatment. Numerous studies have provided direct or indirect evidence supporting that the Akt pathway has profound protective effects against the development of myocardial dysfunction and failure induced by pressure overload^17^,^18^,^19^,^20^,^21^,^22^,^23^,^24^. *In vitro*, we showed that blocking Akt activation diminished the anti-apoptotic effects of TBHQ in stressed myocytes. We further showed that blockade of the Akt pathway *in vivo* accelerated the development of cardiac dysfunction, consistent with several previous studies showing similar deleterious effects of Akt inhibition^19^,^20^,^41^. Moreover, we confirmed the role of Akt in mediating TBHQ effects *in vivo* by demonstrating that the beneficial effects of TBHQ on the cardiac function and apoptosis were lost in the presence of LY294002. On the other hand, the mTOR inhibitor rapamycin did not block the protective actions of TBHQ, indicating that mTOR activation did not have a critical role in TBHQ-induced cardiac protection. It is noted that in the myocardium, the functions of mTOR are controlled by multiple factors in addition to Akt, while Akt activation can also regulate various mTOR-independent pathways^42^. We propose that this may explain the differential outcomes of global inhibition of the Akt pathway and specific inhibition of mTOR in chronic overload-induced cardiac dysfunctions. Based on these data, we suggest that acute activation of the Akt pathway may have a pivotal role in mediating the anti-apoptotic and other protective actions of TBHQ in the heart. It has also been demonstrated that TBHQ may induce the expression of ATF3 (activating transcription factor 3) in the heart, which exhibits beneficial effects in stressed myocytes^13^. In this study, however, the cardiac protective effect of TBHQ was not examined in the absence of ATF3 function. Hence, further studies are needed to define the precise role of ATF3 in this process.

A major mechanism by which Akt produces anti-apoptotic effects is the phosphorylation of the Bcl-2 family protein Bad^43^. In agreement with this, we showed that Bad phosphorylation was also enhanced in TBHQ-treated myocardium. Of note, the overall number of apoptotic cells in LV was relatively small; hence the anti-apoptotic effect of Akt might not fully explain the improvement in the cardiac function induced by TBHQ. We suggest that other mechanisms such as direct inotropic regulating actions of Akt are likely to be involved also^44^,^45^,^46^. Indeed, we found that the endogenous Akt phosphorylation level was enhanced in TAC animals at 2 weeks, which might represent an acute compensatory mechanism at the early stage of cardiac injury. We also found that the level of apoptosis was not significantly different between sham and TAC at 2 weeks, which was consistent with the enhanced Akt phosphorylation and preserved cardiac functions at this time point. A beneficial role of acute activation of Akt in preservation of normal cardiac functions and prevention of myocyte apoptosis under stress conditions has been reported in several experimental studies^47^. In addition, disruption of the Akt downstream signaling impaired the development of compensatory myocyte hypertrophy and functional adaptation to stress stimuli in the heart^24^,^48^, whereas enhanced Akt signaling might contribute to improvement of cardiac functions in failing hearts^49^. Taken these results together, we suggest that pharmacological interventions targeting the Akt pathway may be useful in prevention of the development of myocardial decompensation and failure. On the other hand, we found that TBHQ had no significant effects on the LV geometry or contractility in sham animals, although TBHQ also increased Akt phosphorylation in non-stressed myocardium (data not shown). These data are consistent with those reported by others^13^. It is suggested that during the time window of treatment (4 weeks), the level of Akt activation induced by TBHQ alone was not sufficient to induce hypertrophy or influence the function in normal myocytes in the absence of stress stimuli.

Supporting our *in vivo* findings, we also demonstrated that TBHQ triggered significant Akt activation in cultured myocytes. Both western blot and activity assays on caspase activation showed that TBHQ suppressed oxidant-induced apoptotic response, an effect that was blunted by the phosphatidylinositol 3-kinase (PI3K) inhibitor wortmannin or the Akti, confirming that the anti-apoptotic effect of TBHQ was dependent on the Akt pathway. The mechanism(s) by which TBHQ stimulates Akt phosphorylation in myocytes is not understood. Akt activation is controlled by PI3K, which is a SH2 domain-containing effector downstream of the receptor type protein tyrosine kinases^50^. We showed that TBHQ-stimulated Akt phosphorylation was sensitive to PI3K inhibition, indicating that TBHQ was likely to work upstream of PI3K. Likewise, it was observed that in rat hepatoma cells, TBHQ treatment resulted in an increase in PI3K activity^25^. However, these data cannot determine whether TBHQ directly modulates PI3K enzymatic activity or affects the upstream tyrosine phosphorylation events leading to PI3K activation. Hence, more studies are required to further delineate the molecular mechanisms of TBHQ-triggered Akt phosphorylation.

TBHQ is a lipid-soluble antioxidant with potent inhibitory effects on reactive oxygen-mediated lipid peroxidation and production of reactive aldehyde^7^. We showed that TBHQ treatment decreased the level of 4-HNE and protein carbonylation in the stressed myocardium. Reactive aldehyde-mediated protein carbonylation may have critical roles in the pathogenesis of a variety of human diseases^51^. In cardiac myocytes, it has been shown that protein carbonylation may contribute to alterations in intracellular calcium handling and development of myocyte dysfunction^52^,^53^. Moreover, treatment with carbonyl scavengers such as carnosine exhibited beneficial effects in rat hearts following ischemia-reperfusion injury^54^. On the other hand, exogenous 4-HNE worsened ischemia-reperfusion injury in isolated myocytes^55^. Consistently, we showed that 4-HNE could trigger myocyte apoptosis *in vitro*, a phenomenon which was previously detected in leukocytes^29^. Based on these findings, we suggest that inhibition of reactive aldehyde formation by TBHQ may also underlie its anti-apoptotic and myocardial protective actions.

In summary, we have provided evidence showing that oral treatment with TBHQ significantly inhibited myocyte apoptosis and prevented the development of ventricular dilatation and cardiac dysfunction induced by chronic pressure overload. Supported by other studies^17^,^18^,^19^,^20^,^21^,^22^,^23^,^24^, we suggest that these effects of TBHQ are likely to involve acute activation of the Akt pathway. However, Nrf2-dependent mechanisms are unlikely to have a major role. In addition, TBHQ inhibited formation of reactive aldehyde species, an effect that might also contribute to cardiac protection. Agents that have anti-apoptotic actions are thought to be promising candidates for clinical treatment of heart failure^56^. Although it is recognized that timely activation of the cytoprotective Akt pathway in stressed myocardium may be an effective intervention to delay the development of heart failure, a suitable pharmacological method for this purpose is still unavailable^47^. Our data have provided evidence suggesting that TBHQ may represent a possible candidate.

## Materials and methods

### Animals

All *in vivo* experiments were performed in accordance with the Guide for Care and Use of Laboratory Animals (NIH, 1996) and the ARRIVE (Animals in Research: Reporting *in vivo* Experiments) guidelines, and approved by the Animal Ethics Committee of Qilu Hospital. Male C57BL/6 mice were purchased from Vital River Laboratories (Beijing, China) and maintained on a normal chow diet and water which were provided *ad lib*.

### Transverse aortic constriction

The cardiac pressure overload model was created by transverse aortic constriction (TAC) using a modified surgical technique that did not require thoracotomy^57^,^58^. Briefly, mice of 8 weeks of age were anesthetized with pentobarbital sodium (60 mg/kg, i.p.). A horizontal incision was made at the level of suprasternal notch. Once the trachea was located, a longitudinal cut was made down under the sternum and the aortic arch exposed. Then the aortic arch was partially ligated against a 27-gauge needle using a 7-0 silk suture between the origins of innominate and left carotid arteries. The needle was then retrieved and the incision closed. Sham operation was performed with the same procedure except for the step of aortic ligation. Mice were allowed to recover on a warming pad and a dose of analgesic (Carprofen of 10 mg/kg subcutaneously) was given immediately after awakening. All animals were euthanized 4 weeks after TAC and samples collected for histological and biochemical analyses, unless other time points were included as indicated separately.

### *In vivo* drug treatment

TBHQ powder was purchased from Merck Millipore (Darmstadt, Germany) and directly mixed with the animal food powder at a ratio of 1% (w/w) as described previously^8^. The efficacy of this protocol for TBHQ treatment *in vivo* has also been validated by other groups^11^,^59^,^60^. TBHQ treatment was continued for 4 weeks after the TAC surgery.

### Echocardiographic measurements

Ultrasound echocardiography was performed before and at 2 and 4 weeks after TAC. Under general anesthesia with 1–2% isoflurane, the procedure was conducted using a high resolution echocardiography system (Vevo 770, Visual Sonics, Canada) with a 35-MHz transducer. The heart rate was maintained at ~400 beats/min during the examination by finely adjusting the concentration of the inhalant anesthetic. Two-dimensional parasternal long- and short-axis images of the LV were recorded. Using the M-mode function, the LV end-diastolic diameter (LVDd), LV end-systolic diameter (LVDs), inter-ventricular septum end-diastolic thickness (IVSTd) and LV posterior wall end-diastolic thickness (PWTd) were measured. LV fractional shortening (FS) was calculated as FS (%) = (LVDd- LVDs)/LVDd × 100. Corrected LV mass was derived as described^61^. Parameter measurements were carried out by an independent reviewer.

### Cell culture and treatments

Left ventricles were minced and digested with collagenase II at 37 °C. Released cells were collected by centrifugation and re suspend in a plating medium (80% DMEM/20% M199) containing 10% horse serum and 5% fetal bovine serum. Cells were placed in uncoated culture dishes and allowed to stand for 120 min at 37 °C in the incubator to remove nonmyocytes. Then the unattached myocytes were removed and seeded in collagen-coated dishes, and cultured in a maintenance medium (80% DMEM/20% M199) containing 5% horse serum and 1% fetal bovine serum.

H9c2 rat myocyte-like cells were obtained from ATCC (Manassas, VA, USA) and cultured in Dulbecco’s Modified Eagle Medium supplemented with 10% fetal calf serum, 100 U/ml of penicillin and 100 μg/ml of streptomycin, at 37 °C in a humidified atmosphere with 5% CO_2_. Cells cultured to 80–90% confluence were used for experiments. The following agents were used for cell treatments: TBHQ, 4-hydroxy-2-nonenal (4-HNE) (from Cayman Chemicals, Ann Arbor, MI, USA), wortmannin (from Merck Millipore) and Akt1/2 Kinase Inhibitor (Akti) (from Sigma, St. Louis, MO, USA).

### Histology and immunohistochemistry analyses

Hearts were perfuse-fixed *in situ* at 100 mmHg for 5 min and further fixed in 4% paraformaldehyde overnight, dehydrated, and embedded in paraffin. Short-axis sections of 5 μm thickness were all cut at the horizontal plane at the level of the papillary muscle as described^34^. Sections were stained with hematoxylin and eosin (H&E), picrosirius red, or Masson’s trichrome. For measurements on myocyte cross-sectional area, 100 cells in 5 random 400× fields from each heart were analyzed. Immunohistochemical staining for 4-HNE was performed using a rabbit polyclonal antibody (from Abcam, Cambridge, UK) and VECTASTAIN Elite ABC Kit (from Vector Laboratories, Burlingame, CA, USA). Negative control slides were included in all staining experiments by replacing the primary antibody with non-immune IgG to exclude the presence of non-specific background. To quantify the histological and immuno-stained images, we randomly selected 10 fields for each sample, and used Image-Pro Plus software (Media Cybernetics, Bethesda, MD, USA) for morphometric analysis. The software was operated by an independent investigator in a blind manner.

### Hydroxyproline assay

Hydroxyproline assay was performed to quantify the collagen content in LV as used previously^62^, using a commercial kit from Jiancheng Bioengineering Institute (Nanjing, China) according to the manufacturer’s instructions.

### Western blotting

Western blotting was performed as described^63^. The following primary antibodies were used: Nrf2, AMP-activated protein kinase α (AMPKα), Bad (all from Abcam), Akt, p-Akt, extracellular signal-regulated kinase (ERK), p-ERK, caspase 3, cleaved caspase 3, p-AMPKα (all from Cell Signaling Technology, Beverley, MA, USA), and p-Bad (from Novus Biologicals, Littleton, CO, USA). The Image J software (NIH) was used for densitometry measurement of western blots, which was operated by an independent investigator in a blind manner.

### Assessment of protein carbonylation

The level of protein carbonylation was assessed using a method described previously with some modifications^64^. Protein homogenates of the left ventricular tissue were prepared in cold lysis buffer (100 mM NaCl, 50 mM Tris, pH 7.5, 2 mM EDTA and 1% Triton X-100) supplemented with a protease inhibitor cocktail (Roche, Mannheim, Germany), and adjusted to a protein concentration of 10 mg/ml. Carbonylated proteins were biotinylated by incubating with biotin-hydrazide (Sigma) of 5 mM in the dark for 2 hr at room temperature, and stabilized by further incubating with NaBH_4_ of 50 mM for 1 hr. Aliquots of the samples were separated by SDS-PAGE and transferred to polyvinylidene difluoride membranes. The membrane was blocked in 1% bovine serum albumin solution for 1 h and incubated in a diluted avidin-biotin horseradish peroxidase solution (prepared using the VECTASTAIN Elite ABC Kit) for 30 min with gentle agitation. Biotin-labeled proteins were detected with chemiluminescence (ECL Prime reagent from GE, Piscataway, NJ, USA). Samples prepared without biotin-hydrazide were used as negative control for endogenous biotinylated proteins in the myocardium.

### TUNEL assay

Apoptosis was detected by terminal deoxynucleotidyl transferase dUTP nick end labeling (TUNEL) technique using an ApopTag Plus Peroxidase *in situ* Apoptosis Detection Kit (from Millipore, MA, USA) according to the manufacturer’s instructions. TUNEL positive cells were surveyed in 10 random 400× fields for each section.

### Real time quantitative PCR

Real-time PCR was carried out using Taqman Gene Expression primer-probe sets as described^65^. The 18S RNA was used as the house-keeping gene. The relative quantification method was used for data analysis. Briefly, ΔCt was calculated as the Ct value of the test gene minus that of the house-keeping gene. ΔΔCt was calculated as the ΔCt value of a given gene in treated groups minus that in control group. The relative gene expression level (fold) was expressed as the calculated value of 2^−ΔΔCt^.

### Measurement of caspase 3/7 activity

Caspase 3/7 activities were measured as described using the Caspase-Glo 3/7 Assay kit (Promega, Madison, WI, USA)^65^.

### Statistical analysis

Data were expressed as mean ± standard error of the mean (SEM). For statistical analysis, unpaired *t*-test or one-way analysis of variance (ANOVA) followed by Newman-Keuls multiple comparisons as appropriate were performed using SPSS 13.0 software (SPSS Inc, Chicago, Illinois, USA). A value of *P* < 0.05 was considered statistically significant.

## Additional Information

**How to cite this article**: Zhang, Y. *et al.* The antioxidant compound tert-butylhydroquinone activates Akt in myocardium, suppresses apoptosis and ameliorates pressure overload-induced cardiac dysfunction. *Sci. Rep.* **5**, 13005; doi: 10.1038/srep13005 (2015).

## Supplementary Material

Supplemental Figures 1-7

## Figures and Tables

**Table 1:** Body weight, heart rate and parameters for liver and kidney functions.

	Sham	TAC	TAC + TBHQ
Body weight (g)	27.2 ± 0.2	26.2 ± 0.6	28.3 ± 0.3
Heart rate* (bpm)	456 ± 22	441 ± 18	452 ± 15
ALT (unit/L)	54.0 ± 9.0	39.0 ± 2.0	44.1 ± 5.7
AST (unit/L)	127.5 ± 14.4	125.0 ± 11.4	103.0 ± 10.9
ALP (unit/L)	86.0 ± 5.9	90.6 ± 3.2	79.2 ± 1.5
BUN (mM)	9.7 ± 0.7	11.3 ± 0.8	11.0 ± 0.2
CK (unit/L)	668.6 ± 41.7	908.5 ± 155.4	624.0 ± 131.7

bpm, beat per minute; ALT, alanine aminotransferase; AST, aspartate aminotransferase; ALP, alkaline phosphatase; BUN, blood urea nitrogen; CK, creatine kinase. Data are mean ± SEM, *n* = 4 (Sham), 15 (TAC) and 15 (TAC + TBHQ). *Heart rate was measured during echocardiography.
