# Supplementary material for: The antioxidant compound tert-butylhydroquinone activates Akt in myocardium, suppresses apoptosis and ameliorates pressure overload-induced cardiac dysfunction
Source: Sci Rep. 2015 Aug 11;5:13005. doi: 10.1038/srep13005 (PMC4531315; doi:10.1038/srep13005)
Supplement: Supplemental Figures 1-7 [file srep13005-s1.pdf]

The antioxidant compound tert-butylhydroquinone activates Akt in myocardium, suppresses apoptosis and ameliorates pressure overload-induced cardiac dysfunction

Authors:

Yongtao Zhang
Fang Fang Liu
Xiaolei Bi
Shuangxi Wang
Xiao Wu
Fan Jiang

Supplemental Figure S1

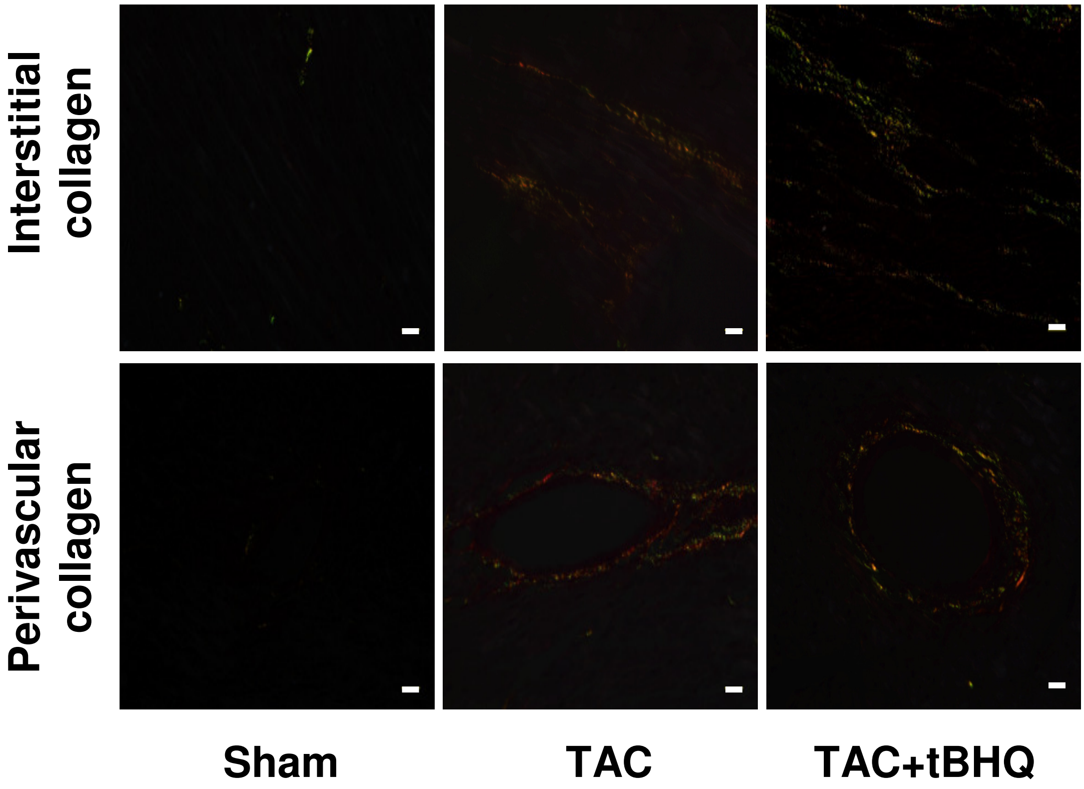

Polarization microscopy images of picrosirius red staining of LV tissues showing the effects of TAC and TAC+tBHQ on the degree of interstitial and perivascular fibrosis. Images were taken with a light microscope (Olympus BX-51). Bar = 20 μm.

Supplemental Figure S2

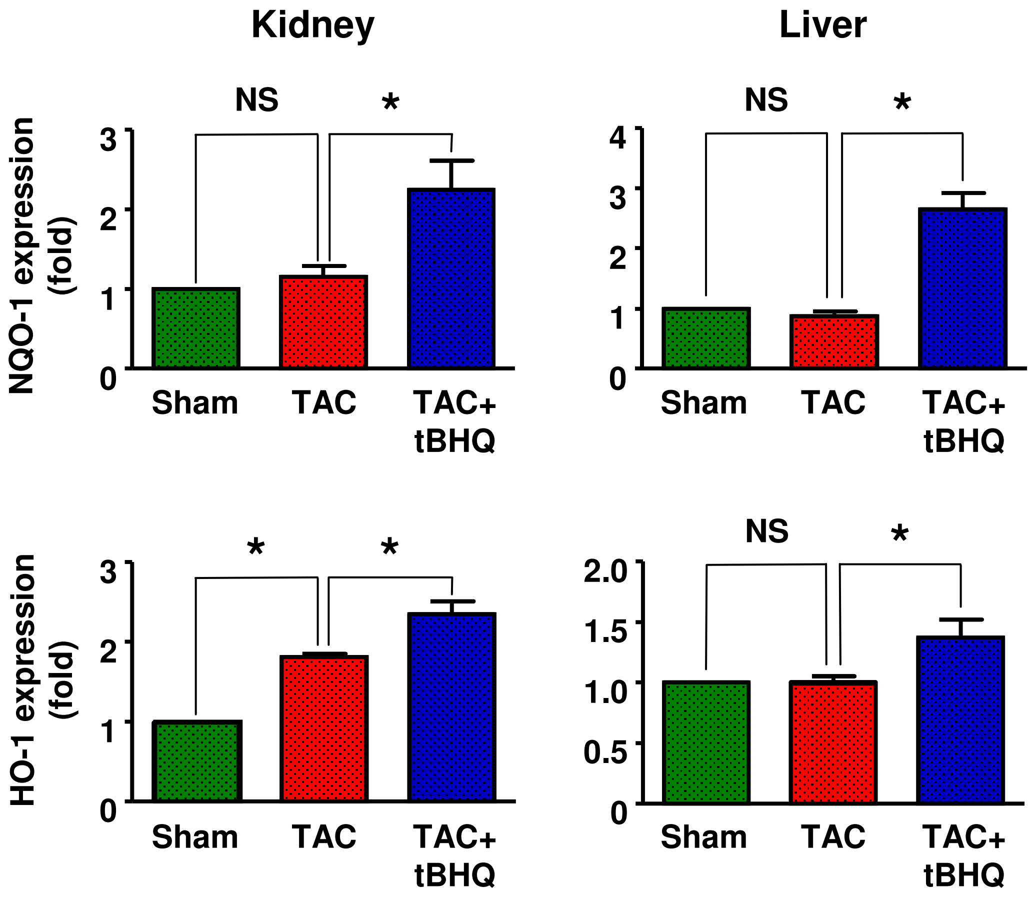

PCR results showing the expression levels of the Nrf2 target genes NQO1 and HO-1 in the kidney and liver in sham, TAC and TAC+TBHQ treated animals. * $P < 0.05$, one-way ANOVA (n = 6). NS, no significance.

Supplemental Figure S3

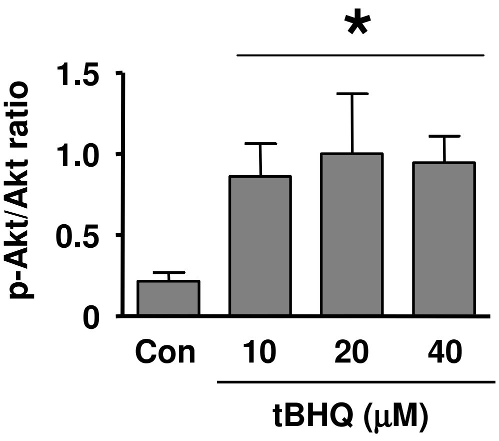

Quantitative densitometry data of the western blot in Figure 8A showing the effects of TBHQ treatment at different concentrations on Akt phosphorylation in cultured H9c2 cells. * *P* < 0.05, one-way ANOVA versus control (*n* = 4).

Supplemental Figure S4

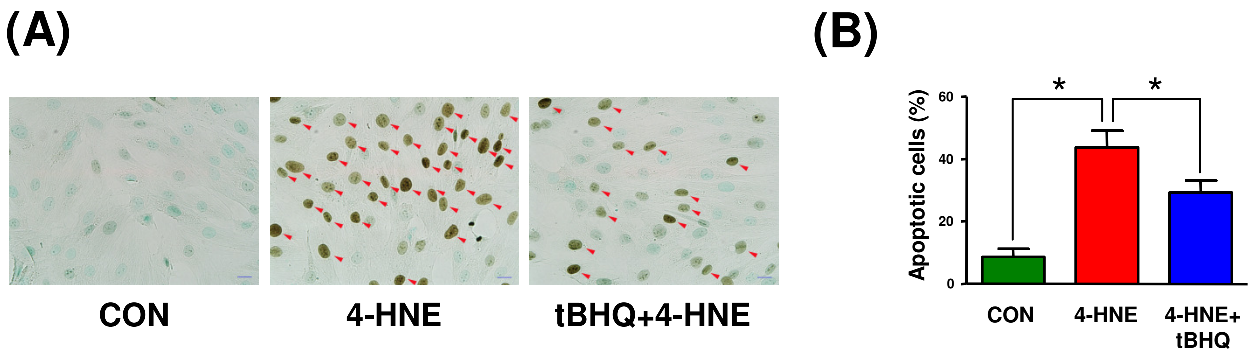

TUNEL staining in cultured H9c2 cells showing the effect of tBHQ (20 μM) pretreatment on 4-HNE (20 μM for 24 hr)-induced apoptosis. (A) Arrow heads indicate the nuclei of apoptotic cells. (B) Cell counting data for (A). * *P* < 0.05, one-way ANOVA, *n* = 3 independent experiments.

Supplemental Figure S5

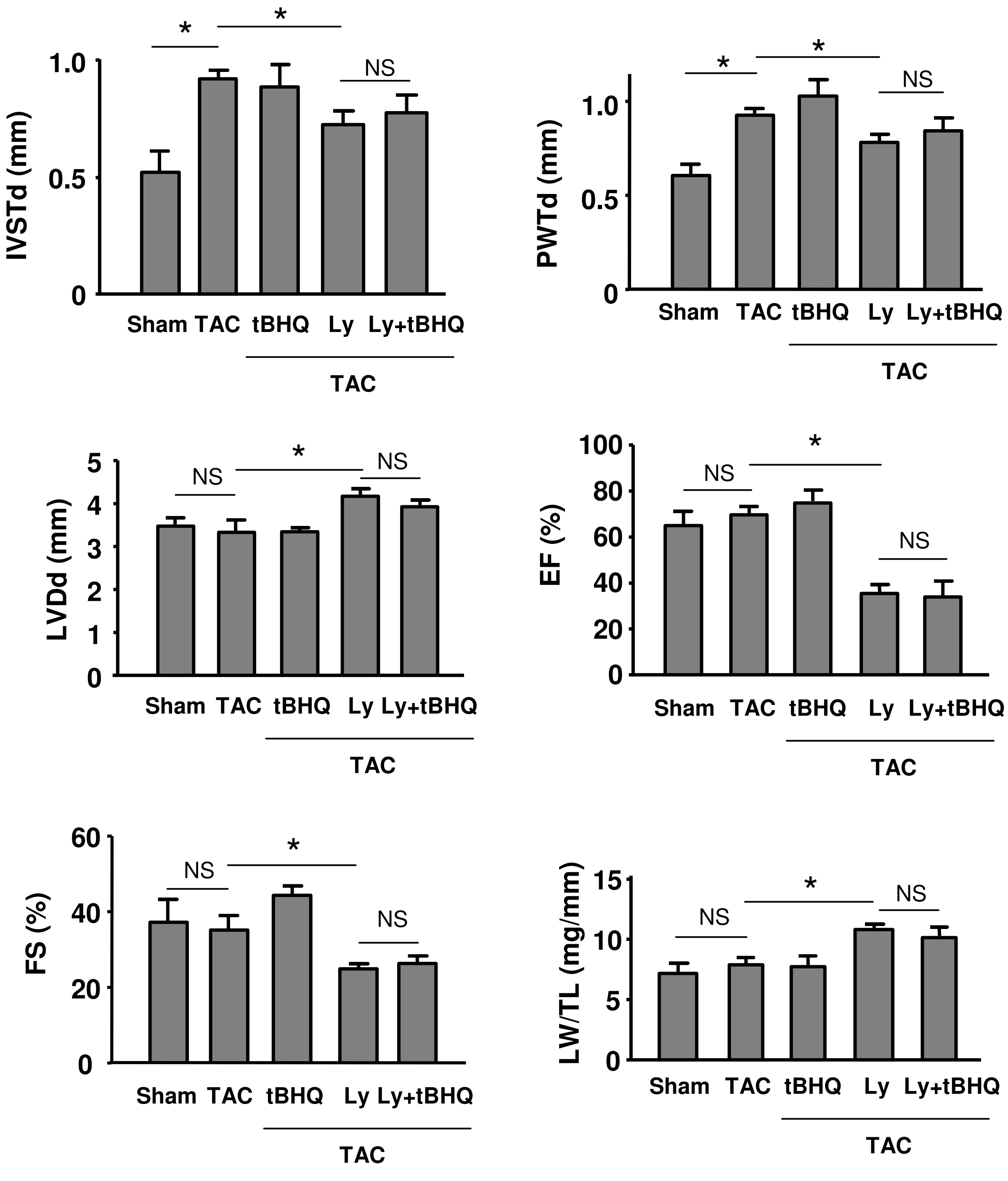

Effects of LY294002 co-treatment in vivo on the actions of TBHQ on echocardiography parameters of cardiac structure and function, and the relative lung weight at 2 weeks after TAC. * *P* < 0.05, one-way ANOVA (*n* = 6). NS, no significance. LW/TL, lung weight to tibia length ratio.

Supplemental Figure S6

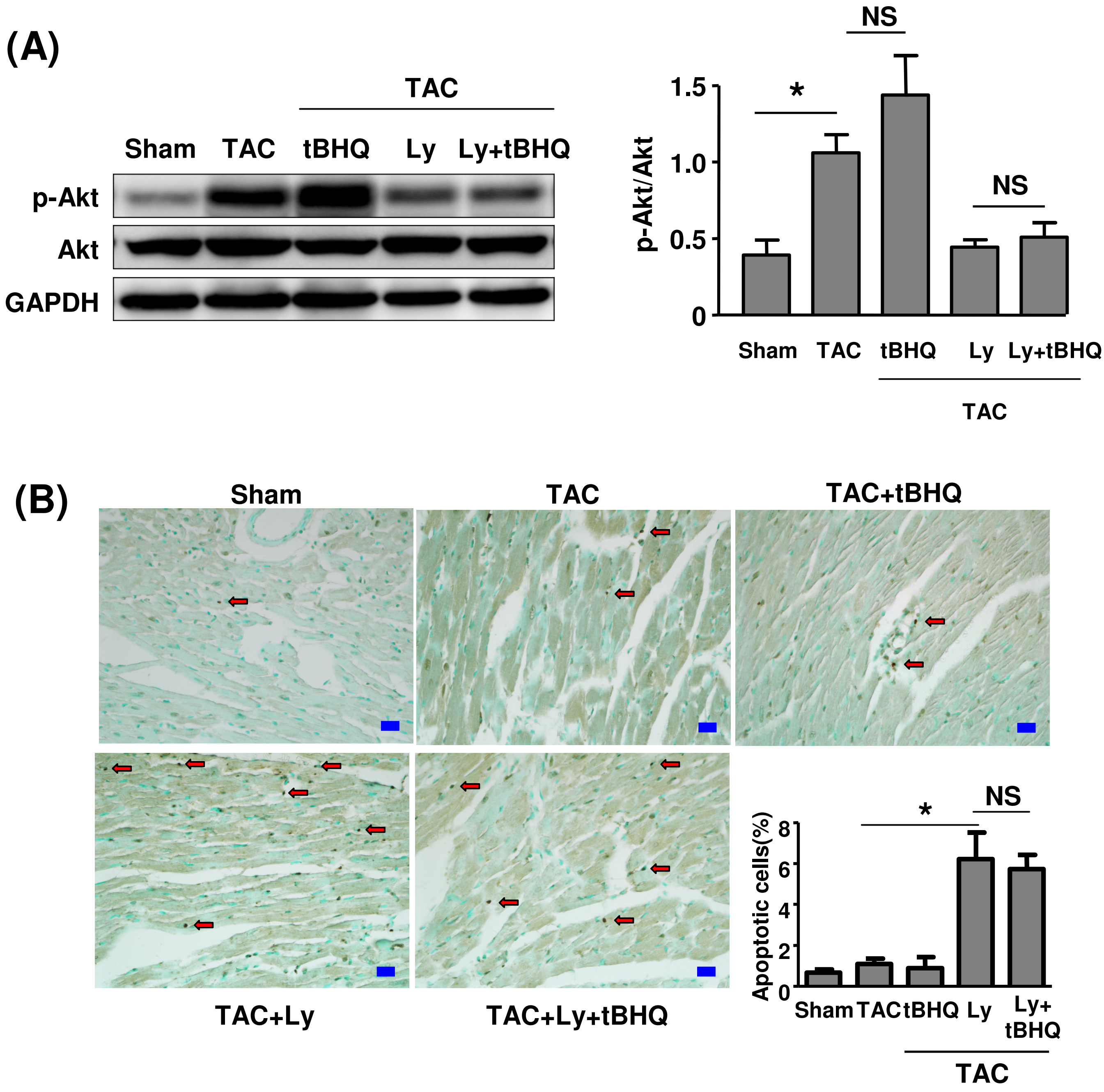

Effects of LY294002 co-treatment in vivo on the actions of TBHQ on (A) Akt phosphorylation level and (B) cell apoptosis at 2 weeks after TAC. * *P* < 0.05, one-way ANOVA (*n* = 6). NS, no significance.

Supplemental Figure S7

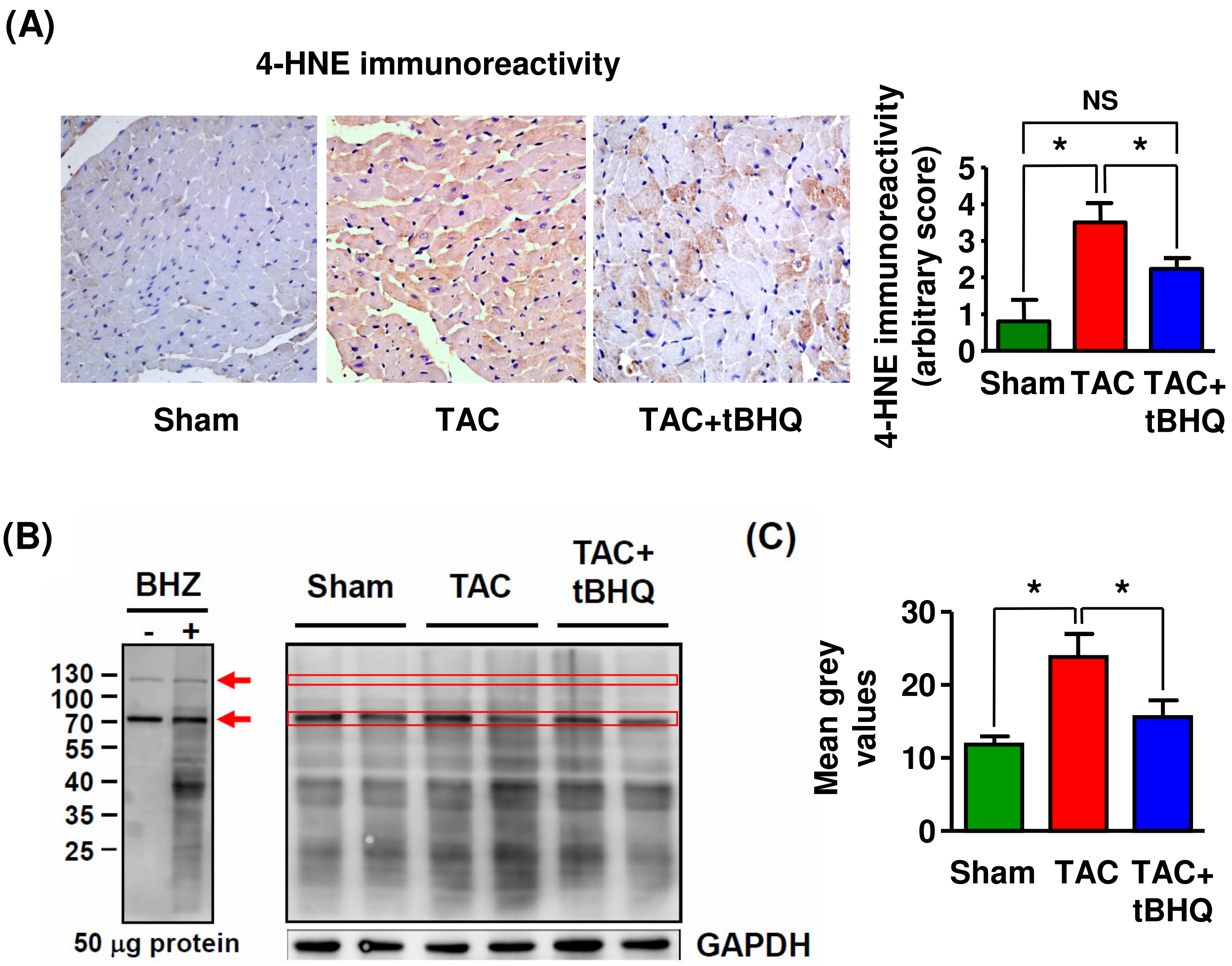

Effects of TBHQ on reactive aldehyde production and protein carbonylation in LV tissues. (A) Immunohistochemistry staining for 4-HNE (brown color) and the corresponding semi-quantitative data. Sections were counter stained with hematoxylin. (B) Carbonylated proteins in the myocardium detected by biotin-hydrazide (BHZ) labeling followed by western blotting. Left panel shows protein samples without and with BHZ labeling. Arrows and boxes indicated the non-specific endogenous biotin-containing proteins. (C) Quantitative analysis of the blots. The mean grey density values for each lane were obtained by selecting the entire area excluding the non-specific signals enclosed by the red boxes, followed by densitometry measurements using Image J software. * *P* < 0.05, one-way ANOVA (*n* = 6).
